# Analyzing the Expression Profile of *AREB/ABF* and *DREB/CBF* Genes under Drought and Salinity Stresses in Grape (*Vitis vinifera* L.)

**DOI:** 10.1371/journal.pone.0134288

**Published:** 2015-07-31

**Authors:** Hana Zandkarimi, Ali Ebadi, Seyed Alireza Salami, Houshang Alizade, Niranjan Baisakh

**Affiliations:** 1 Department of Horticulture, Faculty of Agriculture, University of Tehran, Karaj 31587, Iran; 2 School of Plant, Environmental and Soil Sciences, Louisiana State University Agricultural Center, Baton Rouge, LA 70803, United States of America; University of Delhi South Campus, INDIA

## Abstract

Expression patterns of four candidate *AREB/ABF* genes and four *DREB/CBF* genes were evaluated in leaf and root tissues of five grape varieties (‘Qalati’, ‘Kaj Angoor’, ‘Sabz Angoor’, ‘Siahe Zarghan’, ‘Bidane Safid’) with differential response to drought stress. Among the *AREB/ABF* genes, *AREB1* and *ABF2* showed up-regulation in response to drought stress in leaf and root tissues of all varieties while *AREB2* and *ABF1* showed down-regulation in both leaf and root tissues of the sensitive variety ‘Bidane Sefid’ in response to drought and salt stress. Among the *DREB/CBF* genes, CBF4 was the most responsive to drought stress in both leaf and root tissues. *CBF2* and *CBF3* showed up-regulation in all varieties in response to drought stress in leaf except in ‘Bidane Sefid’. Under salinity stress, *AREB2* and *ABF2* showed up-regulation in response to the increasing level of salinity in the leaf tissues but in the root tissues *ABF2* was up-regulated in response to increasing NaCl concentration while *AREB2* was down-regulated. Therefore, it seems *AREB2* has tissue-specific response to salinity stress. All *CBF* genes were up-regulated in response to salinity stress in the leaf and root tissues. Expression data suggested that *CBF2* is more responsive to NaCl stress. Among all four promising and stress tolerant varieties ‘Siah Zarghan’ and ‘Kaj Angoor’ were more tolerant than ‘Qalati’ and ‘Sabz Angoor’ to drought and salinity.

## Introduction

Plants respond and adapt to stress conditions through different mechanisms at the molecular, cellular, physiological, and biochemical levels. An important step in understanding how a genome functions in a different environmental cue is to determine the pattern as how the expression of the genes is regulated. Various adverse environmental stresses change the expression pattern of a variety of genes in many plant species [1]. Among the stress related genes, transcription factors (TFs) play an important role in regulating plant’s response to stress conditions. TFs act as master switches and trigger simultaneous expression of a large number of stress-response genes that contribute to the stress tolerance phenotype. With the exception of a few TFs that play a role in primary growth processes, most TFs are up-regulated under stress conditions [2][3]. Several families of plant TFs play significant roles in regulation of gene expression under abiotic stresses [4]. Genes are often linked by several binding sites for distinct transcription factors, and different transcription factors coordinately regulate the transcription of these genes.

One of the most important adjustments under stress condition in plant is the change in the level of its phytohormones, such as abscisic acid (ABA). ABA, in addition to playing important roles in many physiological processes such as seed dormancy and development, embryo morphogenesis, leaf senescence and promotion of stomata closure, is considered as a plant stress hormone that is accumulated under osmotic imbalance caused by abiotic stresses. ABA is known to play a key role in stress responses and tolerance in plants. Genes that are altered in their expression in response to ABA accumulation in plant during stress belong to ABA-dependent pathway, and other ones belong to ABA-independent pathway. Although these two pathways are distinct, crosstalk between the two pathways are documented in several plants [5][6].

ABA perceives stress uniquely and acts as an endogenous messenger in plant cells to induce a double negative regulatory pathway in which ABA is bound to the ABA receptors RCARs/PYR1/PYLs (Regulatory Components of ABA Receptor/Pyrabactin Resistance Protein1/PYR-Like proteins) to form the complex that provides an active site for the PP2Cs (type 2C protein phosphatases). This causes inhibition of the activity of PP2C as a negative regulator of the pathway which leads to induction of SnRK2 as a positive regulator of downstream signaling and subsequent phosphorylation of the target proteins [7][8]. Thus, in the presence of ABA, the *PP2C*s are inactivated to repress *SnRK2* phosphatase activity and then *SnRK2* could initiate ABA-responsive regulation pathway and activate the most significant cis-element ABA-responsive element (ABRE) to regulate the expression of many genes under osmotic stress conditions. Various TFs regulate the expression of ABA-responsive genes through ABRE, which contains the core sequence PyACGTGGC that regulates dehydration- and high salinity-responsive gene expression in plants in the ABA-dependent pathway. ABRE-binding protein (AREB)/ABRE binding factors (ABFs), as a member of basic domain leucine zipper (bZIP) TF, are activated in the presence of ABA and regulate the expression of genes by binding to the ABRE in their promoter regions. Several studies showed that exogenous application of ABA induced a number of genes that respond to dehydration and cold stress in the ABA-dependent pathways [5][9]. On the other hand, several studies reported induction of *AREB1*, *AREB2* and *ABF3* under drought, high salinity and ABA treatments in plants [9–11]. Previous studies showed that of the nine members of AREB/ABFs in *Arabidopsis*, *AREB1/ABF2*, *AREB2/ABF4*, and *ABF3* were induced by ABA and osmotic stress in vegetative tissues [12–16], which suggested that they are involved in ABA and/or stress signaling. In rice, *OsABF2* was shown to be induced by drought, salinity, and ABA treatment [10]. Fujita et al. [15] reported that transgenic *Arabidopsis* plants overexpressing the active form of *AREB1* showed ABA hypersensitivity and enhanced drought tolerance. Overexpression of *ABF2* in *Arabidopsis* enhanced its tolerance to drought, high salt, heat and oxidative stresses [17]. Lettuce and *Agrostis mongolica* transgenic plants ectopically expressing ABF/AREB members were more tolerant than wild type to drought and cold/heat stresses [18][19]. Similarly, overexpression of the *PtABF* gene of *Poncirus trifoliata* enhanced dehydration and drought tolerance in tobacco [20]. These studies validated the significance of ABF/AREB family members in plant’s response to various environmental stresses [21]. Yeast two-hybrid screening in grape (*Vitis vinifera*) identified positive interactions between two *VvABFs* and *VvSNRK2* [11]. The authors reported that *VvABFs* expression under various abiotic stresses was tissue-specific where *VvABF1* was highly induced in roots and *VvABF2* was up-regulated in the leaves under drought, salt and ABA treatments.

Some of the most important TFs in the ABA-independent pathway that play significant roles in responses to important abiotic stresses include dehydration-responsive element binding protein (DREB)/C-repeat binding factor (CBF), NAC (NAM, ATAF, and CUC) and MYB/MYC [22–24]. The core sequence for dehydration-responsive element (DRE) is A/GCCGAC, which is a Cis-acting element for regulation of cold and dehydration-responsive gene expression in plant [22–26]. DREB/CBFs were known as low temperature responsive genes but their expression pattern in some plants were changed in response to different stress conditions. For example, *AtCBF4* was responsive to drought stress, *OsDREB1A* was induced in response to salt stress, and *AtDREB1D*, *VvCBF1*, *VvCBF2* and *VvCBF3* were responsive to ABA [22][27][28].

In grape genome, Xiao et al. [28] identified three *CBF/DREB1* genes; the deduced proteins were 42–51% identical to AtCBF1 and contain CBF-specific amino acid motifs, and were responsive to low temperature. The expression of these genes also changed under different levels of drought stress and ABA treatment. In another study [29], the authors also reported that the expression of *CBF4* genes in *V*. *vinifera* and *V*. *riparia* was regulated in response to low temperature, drought, and salinity. Over-expression of *AtCBF3* increased stress tolerance in transgenic *Arabidopsis*, whereas overexpression of the tomato *LeCBF1* did not have the same effect in transgenic tomato [30]. ABA-mediated induction of *DREB/CBF* genes has also been identified in some plants. The crosstalk among ABA-dependent and ABA-independent stress response pathways and TF families could be because of the commonality in plant’s responses to different stresses, such as salinity and cold stresses, which create osmotic stress that crosstalk with drought stress [22][27][28][31–34].

Grape is an economically important most widely cultivated woody fruit crop in the world. Viticulture is closely associated with Iran history as the center of origin of *V*. *vinifera*. Traditional rainfed cultivation of grape in different edaphoclimatic zones of Iran shows that some genotypes have evolved greater tolerance than others to stress conditions, especially drought and salinity [35][36]. Considering the fact that *AREB/ABFs* and *DREB/CBFs* are major transcription factors that respond to abiotic stresses by regulating downstream genes stress-responsive genes in ABA-dependent and independent pathways, the present study was undertaken to determine the expression pattern of these genes in four promising grape varieties of Iran in response to drought and salinity stress.

## Materials and Methods

### Plant material and stress treatments

Cuttings of four promising drought and salt tolerant varieties [‘Qalati (Q)’, ‘Kaj Angoor (KA)’, ‘Sabz Angoor (SA)’, ‘Siahe Zarghan’ (SZ)] and the white seedless drought and salt-sensitive cultivar ‘Bidane Sefid’ (BS) [37] of grape (*Vitis vinifera*) with winter-dormant buds were obtained in 2012 from the Grapevine Research Station of Takestan- Qazvin, Iran. All of these cuttings were from the middle part of the branch with same diameter (1.0–1.5 cm) with 3–4 dormant buds. Cuttings were rooted in pots filled with sand under greenhouse conditions first, and rooted shoots during spring were planted in 25 cm (height) x 19 cm (dia) pots containing a mixture of soil and composts (1:2) to grow for one year out door. One-year-old rooted plants were pruned to three winter buds and roots of 8 to 10 cm in length in the following winter, and planted in 20 L pots containing approximately 15 L mixture of sand and Garden soil (2:1). Plants were grown out door and irrigated every 3 to 4 d with tap water mixed with one fourth concentration of Hoagland nutrient solution. In 2013 August, which is the warmest month of the year in most parts of Iran with a mean temperature of ~35°C and ~0.64 mm rainfall, seven-month-old uniform plants were selected for stress experiments. For drought stress treatments (S1a Fig), four levels of water stress (well-watered control, -0.3 MPa; medium stress, -0.7 MPa; high stress, -1 MPa; and severe stress: -1.5 MPa) were applied based on curve soil moisture. For salinity stress, two levels (100 mM and 200 mM) of NaCl were applied, and to reduce the negative effects of NaCl on calcium absorption, CaCl_2_ (one tenth of NaCl concentration) was added to the salt solution. Salinity stress was imposed (S1b Fig) with daily watering of salinized plants with 6 L of salt solution throughout the experiment. Salt concentration of the irrigation solution started with 10 mM NaCl in the first day and NaCl concentration increased 20 mM each two days until day 9 (for 100 mM stress level) and day 19 (for 200 mM stress level). Primary experiment showed that 6 L water irrigation each day creates -0.3 MPa suction in the soil, which is same as the control used in the study. Three different plants (biological replicates) were used for each treatment. The leaf and root tissues were harvested individually from each plant in liquid nitrogen at different stress levels for drought and 9 d and 19 d for salt stress treatment and stored in a -80°C freezer until RNA extraction.

### RNA purification and cDNA synthesis

Total RNA was extracted according to Reid et al. [38] using an extraction buffer that contained 300 mM Tris HCL (pH 8.0), 25 mM EDTA, 2 M NaCl, 2% CTAB, 2% PVPP, 0.05% spermidine trihydrochloride, and 2% β-mercaptoethanol. The RNA was further purified using an RNeasy kit (Qiagen, Valencia, CA). First strand cDNA was synthesized from 2 μg of total RNA using the iScript cDNA synthesis kit (Bio-rad, Hercules, CA) according to the manufacturer's instructions.

### Gene Identification and PCR primer design

In order to identify the *V*. *vinifera AREB/ABF* genes, protein sequences of *A*. *thaliana AREB/ABF* genes were used for a BLASTP search against the *V*. *vinifera* genome. The bZIP domain was checked in all the grape candidates. All known *AREB/ABF* gene sequences in the NCBI (http://ncbi.nlm.nih.gov) database along with *A*. *thaliana* genes and grape candidates were clustered (S2 Fig), and based on their classification in the cluster, four grape *AREB/ABF* candidate genes were selected. *DREB/CBF* genes were identified from *V*. *vinifera* genome based on their annotation against the NCBI database. Primers were designed for each of the genes using Primer 3 (http://bioinfo.ut.ee/primer3) and NCBI primer blast (http://www.ncbi.nlm.nih.gov/tools/primer-blast). For family gene distinction, one of the primer pairs was designed from the bZIP domain and the other one was designed from the conserved sequences of the particular gene. Details of the primers are provided in Table 1. Quantitative real time PCR (qRT-PCR) reactions were performed using SYBR Green kit (Bio-rad, Hercules, CA). Reactions were performed in a 20 μl volume containing 3 μl of diluted cDNA (5 times diluted from the first strand cDNA stock) and 10 μl 2× iQ SYBR Green Master Mix Supermix as described earlier [39]. Elongation factor (*VvEF1α*; accession no. LOC100261589) was used as the internal reference gene (S3 Fig).

**Table 1:** Primer sequences of the genes used for expression analysis in grape.

Primer	Sequence (5’– 3’)
VvAREB1-F	CTTCCATATACTCCTTGACC
VvAREB1-R	AGGCAATGTCAAAGAACCC
VvAREB2-F	TAACCACATTAGCAACTCCC
VvAREB2-R	CATTATGAACGCTGTCCTGC
VvABF1-F	TGATAAACACATGGCTGACC
VvABF1-R	TCTTCCAAAGTCATCTCCCC
VvABF2-F	GCAGGTGTGATTAGTTTAGGG
VvABF2-R	CTTGAGTCCAACACTCCTGG
VvCBF1-F	AGAGAAGGTTGGAGATGGTTCA
VvCBF1-R	CAGGTGGAGTAAGGAGCAAAC
VvCBF2-F	CTGCTTCTTCCGACTCTC
VvCBF2-R	GCACTTCACTCACCCATTTGTT
VvCBF3-F	AAGTGCGGGATCCCAAAACC
VvCBF3-R	GGAGTCGGGGAAATTGAGC
VvCBF4-F	ACCCTCACCCGCTCGTATG
VvCBF4-R	CCGCGTCTCCCGAAACTT
VvEF1a-F	CGGGCAAGAGATACCTCAAT
VvEF1a-R	AGAGCCTCTCCCTCAAAAGG

## Results

Eight genes, four each belonging to *AREB/ABF* and *DREB*/*CBF*, in five grape varieties showed time- and tissue-dependent variation in their expression patterns in the leaf and root tissues under drought (Figs 1 and 2; S1 and S2 Tables) and salt (Figs 3 and 4; S3 and S4 Tables) stress treatments.

**Fig 1:**
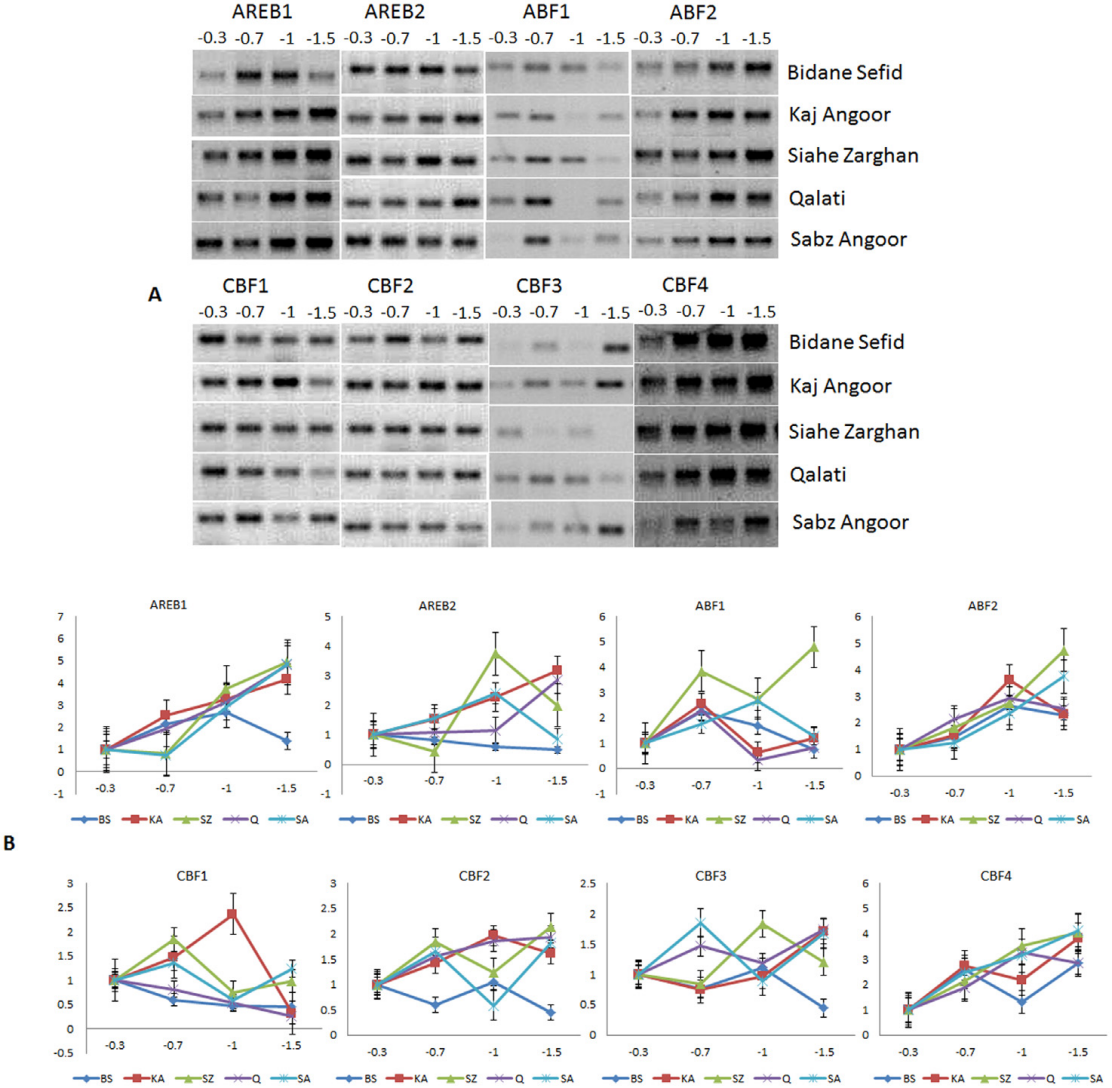
Semiquantitative (A) and Quantitative (B) expression analysis of *AREB/ABF* and *DREB/CBF* genes in the leaf tissues of five different varieties of grape under drought stress.

**Fig 2:**
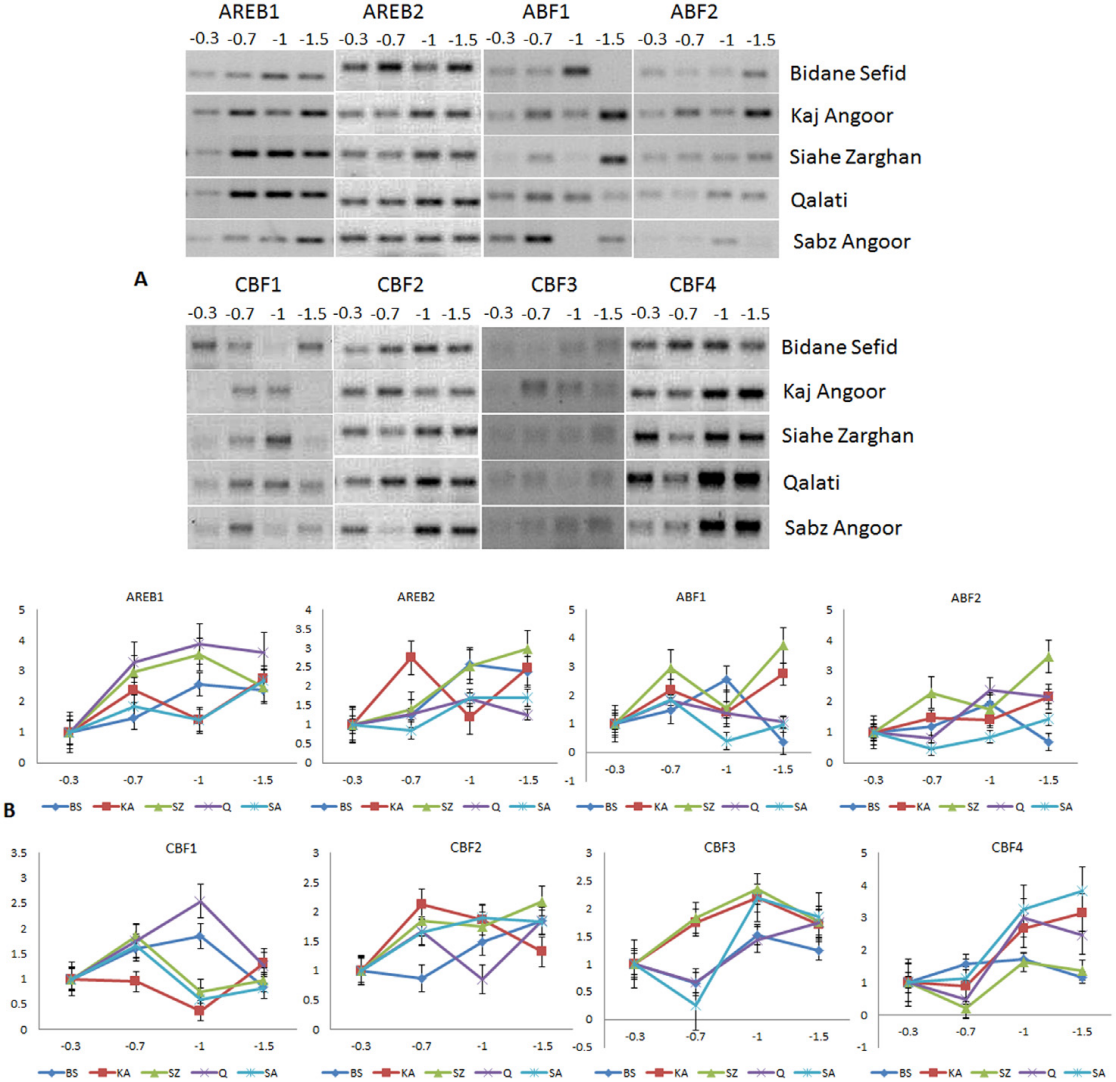
Semiquantitative (A) and Quantitative (B) expression analysis of *AREB/ABF* and *DREB/CBF* genes in the root tissues of five different varieties of grape under drought stress.

**Fig 3:**
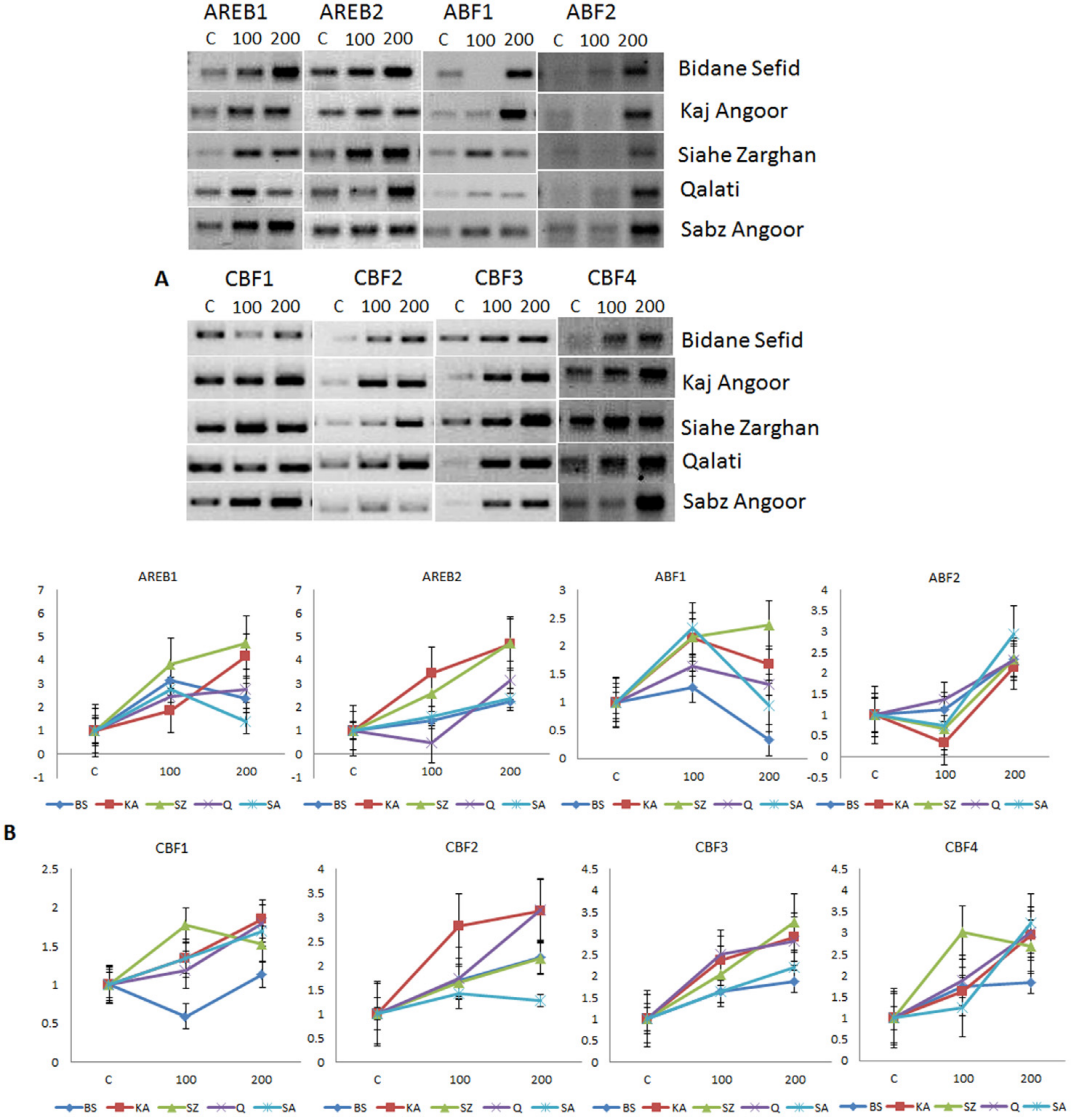
Semiquantitative (A) and Quantitative (B) expression analysis of *AREB/ABF* and *DREB/CBF* genes in the leaf tissues of five different varieties of grape under salt stress.

**Fig 4:**
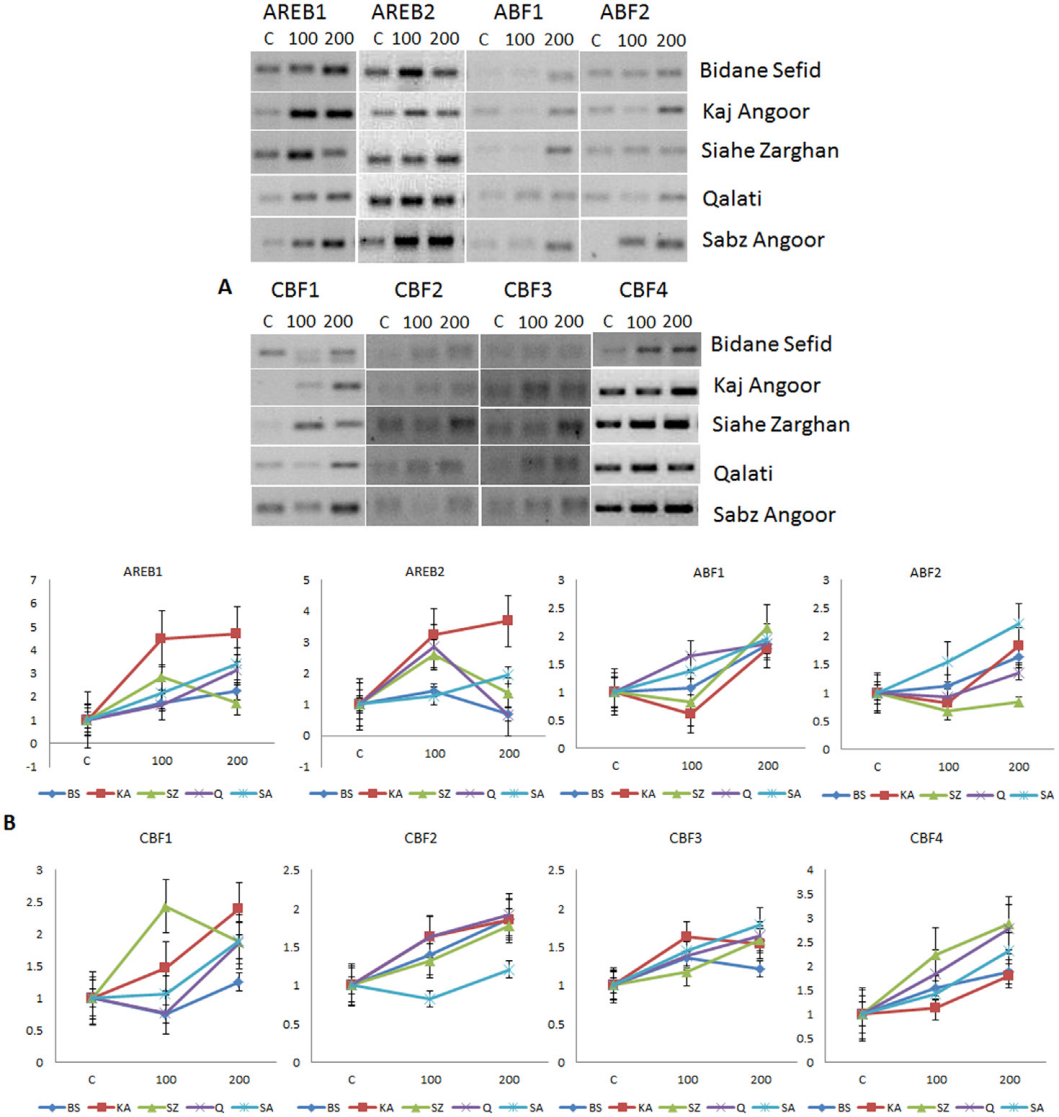
Semiquantitative (A) and Quantitative (B) expression analysis of *AREB/ABF* and *DREB/CBF* genes in the root tissues of five different varieties of grape under salt stress.

### Expression of *AREB/ABF* genes under drought

Under drought stress, *VvAREB1* gene in grape, a homolog of *AREB1/ABF2*, was significantly up-regulated in the leaf of all genotypes at high (-1 MPa) and severe (-1.5 MPa) stress levels, although its expression in BS under severe stress was lower than that under moderate and high drought (Fig 1; S1 Table). The gene showed an incremental up-regulation along with the severity of stress level in KA and Q. In SZ and SA, the gene showed down-regulation under medium stress level following which its expression was upregulated. In the root tissue (Fig 2; S2 Table), the gene showed up-regulation under drought stress in all five varieties. The expression of *AREB1* was similar in the leaf and root tissues in BS where the level of induction across different stress levels was, in general, lower than the four resistant varieties.

*VvAREB2* was closer to *AREB1*/*ABF2* in the cluster (S2 Fig); its transcript accumulation, like *AREB1*, showed an increasing trend of up-regulation with the severity of drought stress in the leaf of KA and Q. In SZ and SA, the transcript accumulation reached its peak at the high level drought stress. However, it was down-regulated in SZ immediately after imposition of low level of stress, whereas it was down-regulated in SA at the severe drought condition. *VvAREB2* showed down-regulation under drought in the leaf of the sensitive variety BS, but the level of its expression was higher compared to the control condition of other varieties. However, in the root, it was up-regulated in BS, Q, and SA under drought stress, although with a slight reduction in BS and Q at severe stress and in SA at the low level stress. On the other hand, SZ showed increased accumulation of *VvAREB2* mRNA with increasing stress level while KA showed an up-regulation compared to the control but the level was highest under low followed by severe level drought stress, and the level dropped at high level stress.

*VvABF1*, the grape homolog of *ABF3* gene, showed a different expression pattern than other *AREB/ABF* genes. In the leaf tissue, all five varieties had increased mRNA abundance under low level drought stress, after which the pattern was variable in the varieties. SZ showed highest up-regulation of *VvABF1* at severe stress level with a slight reduction at high level stress. In SA, up-regulation was observed at all stress levels, although the level of accumulation at severe stress was slightly less than moderate and high level stress. In the leaf of KA and Q, the level of *ABF1* expression, after an up-regulation at moderate level stress, was down-regulated at high level drought and then slightly increased, but the level was still lower than the control. The sensitive variety BS showed a declining trend after 2-fold up-regulation under moderate stress until the level was lower than the control under severe stress. In the root tissue (Fig 2), *ABF1* in BS was down-regulated under severe stress while there was an increasing up-regulation under moderate to high drought stress. SZ and KA showed similar pattern of expression with up-regulation under all levels of stress and a slight decline (still higher than the control) under high level drought stress. The variety Q showed up-regulation of *ABF1* at -0.7 MPa and then the level declined during subsequent levels of stress with down-regulation under severe stress compared to the control. However, *VvABF1* had an up-regulation in its transcript accumulation under moderate stress following which it was down-regulated.

*VvABF2*, the homolog of *AtABF3* gene, was up-regulated in the leaf under stress in all varieties. SZ and SA showed an up-regulation of *ABF2* with the highest mRNA accumulation under severe drought stress, whereas the peak for other varieties was observed at high level drought. The expression level of *ABF2* in the leaf of SZ was significantly higher than other varieties under control condition. However, expression pattern of *ABF2* in the root varied with genotypes. In the drought sensitive variety BS, it was slightly up-regulated under moderate stress followed by a 2-fold increase under high stress level and then significantly down-regulated to an undetectable level under severe drought. It was up-regulated in SZ and SA with highest expression under severe stress, but in SA, it was down-regulated under moderate and high level drought. On the other hand, it had the highest expression under high level of drought stress in Q, with slight down-regulation under moderate stress. The expression pattern of *ABF2* in the leaf and root of BS was same at all stress levels and also in the SA and Q except the medium level in the other stress level has same trend in both leaf and root.

### Expression of *DREB/CBF* genes under drought stress

In addition to *AREB/ABF* genes, four grape genes, showing similarity with *DREB/CBF* genes, were also evaluated for their expression under different levels of drought stress. *VvCBF1* showed down-regulation in its transcript accumulation in the leaf of BS in response to increasing stress level. Same trend was observed in the leaf of variety Q. The gene also showed similar pattern in the leaf of SA and SZ where after an up-regulation at the moderate drought stress, its expression was down-regulated under high level stress and then slightly up-regulated under severe drought. KA showed a different expression pattern; it showed an up-regulation under both moderate and high level stress, but under severe stress level, its transcript accumulation was significantly down-regulated. In the root tissue, SZ and SA showed similar pattern like in the leaf where the gene showed up-regulation under moderate stress level and then down-regulation under high and severe stress conditions. The expression pattern of *VvCB1* was same in the root of Q and BS where it was increasingly up-regulated under moderate and high stress levels and then declined in its transcript accumulation under severe drought stress. On the other hand, in KA it was down-regulated in response to moderate and high levels of drought but was up-regulated under severe stress condition.

*VvCBF2* expression pattern in the leaf of KA and Q was similar where the mRNA content showed up-regulation at all levels of stress but with a slight decline in KA under severe stress. SA and SZ also displayed same trend; up-regulation under moderate and severe stress, and down-regulation under high level of drought. The gene showed significant down-regulation in the sensitive variety BS compared to the control, especially under moderate and severe drought conditions. On the contrary, the expression level of *VvCBF2* in the root of BS was higher than the control under high and severe drought. In the root of KA, Q and SZ, the gene showed varying level of up-regulation under different levels of drought. For example, SA showed increasing *CBF2* mRNA accumulation with increasing stress level; KA had the highest accumulation at moderate drought, SZ and Q under severe drought condition, although *CBF2* was down-regulated under high drought in Q.

In the leaf tissue of BS and SZ, *VvCBF3* showed similar pattern of expression where it showed down-regulation under moderate drought followed by increased transcript accumulation under high and severe stress, but the mRNA content was still lower than the control in BS. Similarly, Q and SA showed similar pattern in the expression of *CBF3*. The gene was up-regulated under moderate stress and, after a brief decline, was up-regulated to its maximum peak. The three varieties Q, SA and BS showed similar trend in the root tissue where the gene showed up-regulation after being down-regulated at moderate stress and the highest up-regulation at high (SA and BS) and severe drought (Q). The expression of *CBF3* in the root of KA and SZ showed up-regulation under all levels of stress, although with a slight decline under severe drought stress.

*VvCBF4* showed up-regulation in its transcript accumulation in response to increasing drought stress in the leaf tissue of all varieties, although at varying level in comparison to the control. Interestingly, the *CBF4* expression level in the leaf was very high under non-stressed control in SZ and KA. All varieties had a higher basal mRNA content of *CBF4* than the sensitive variety BS. Variety Q had its highest peak of *CBF4* under high drought condition, whereas all others had its highest expression under severe drought stress. KA and BS showed a reduction of *CBF4* expression under high stress but the level was still higher compared to the control. Increasing trend of *CBF4* accumulation was observed with increasing severity of drought stress in SA and SZ. In the root of KA and SA, the gene showed up-regulation under high and severe stress, and a slight down-regulation under moderate stress in Q. In the sensitive variety BS, after an up-regulation under moderate and high level drought, the expression of *CBF4* returned to the basal level under severe drought. Expression level of *CBF4* in the root of SZ and SA was higher than other varieties under non-stressed control condition. At the same time, SA and SZ displayed down-regulation of *CBF4* at the primary stage (moderate level) of drought stress.

### Expression of *AREB/ABF* genes under salt stress

Expression profiles of the *AREB/ABF* genes in all the five varieties were also evaluated under two salinity levels, 100 mM and 200 mM NaCl. Like drought stress, the genes showed temporal and spatial variation in their expression pattern under salinity (Figs 3 and 4). *AREB1* was up-regulated in the leaf tissues of all the varieties under both levels (100 mM and 200 mM NaCl) of salinity stress as compared to the unstressed control (Fig 3). However, at 200 mM salinity level, its expression in KA, SZ and Q was higher in comparison to that under 100 mM NaCl, whereas its expression level declined in BS and SA at 200 mM compared to 100 mM. In the root tissue (Fig 4), *AREB1* was up-regulated in all the varieties under both levels of salinity except SZ, where the level was higher at 200 mM NaCl in comparison to 100 mM NaCl. *AREB1* expression was highest in SZ (4.7-fold) in the leaf under 200 mM NaCl, but KA maintained its high expression of 4.2-fold and 4.7-fold in leaf and root tissues at 200 mM salinity level, respectively, in comparison to other varieties.

*AREB2* expression in the leaf tissue of all the varieties was up-regulated with increasing salt stress level, although in the variety Q, down-regulation was observed at 100 mM salinity level. In the root tissue, *AREB2* was up-regulated in all the varieties under 100 mM NaCl. But, under 200 mM NaCl, KA and SZ maintained *AREB2* abundance while Q and BS showed down-regulation, and SZ showed decreased level of up-regulation in comparison to 100 mM NaCl. The varieties KA and SZ behaved similarly for both *AREB1* and *AREB2*.

*ABF1* was up-regulated under 100 mM NaCl in the leaf of all the varieties, but under 200 mM NaCl, BS showed 0.3-fold down-regulation while SA was slightly down-regulated (0.9-fold) as compared to the control. SZ showed an increased accumulation of *ABF1* transcript, whereas KA and Q showed its up-regulation at a lower level than 100 mM NaCl. In the root tissues, *ABF1* was down-regulated in KA and SZ at 100 mM salinity level, but remained unchanged in BS, whereas in SA and Q, it was up-regulated. With increasing salinity level (200 mM NaCl), all the varieties showed up-regulation of *ABF1*.

*ABF2* was down-regulated in the leaf tissues of KA, SZ and SA at 100 mM NaCl. On the other hand, it remained almost unchanged in BS, while it was up-regulated in Q. Under 200 mM NaCl, its mRNA content was up-regulated in the leaf tissues of all varieties with the level being highest in SA (2.9-fold). In the root tissues, *ABF2* was up-regulated under 200 mM NaCl in all varieties except SZ, which showed down-regulation under both 100 and 200 mM NaCl. At 100 mM salinity level, except SA and BS, which showed *ABF2* up-regulation, other varieties showed down-regulation as compared to the control.

### Expression of *DREB/CBF* genes under salt stress

Under both levels of salinity, *CBF1* mRNA accumulation was up-regulated in the leaf tissues of all varieties except BS, which showed its down-regulation at 100 mM salinity. SZ showed a slightly reduced level of *CBF1* mRNA under 200 mM NaCl stress as compared to 100 mM NaCl. *CBF1* expression pattern in root was similar to leaf, where BS and Q showed *CBF1* down-regulation at 100 mM salinity level and the expression level did not change in SA. The transcript accumulation of *CBF2* was up-regulated under both 100 mM and 200 mM NaCl in the leaf and root tissues of all varieties, except in SA where it was down-regulated in root tissue at 100 mM salinity as compared to the control, and showed a slight decline in *CBF1* mRNA at 200 mM compared to 100 mM NaCl. Variety Q had the highest abundance of *CBF2* mRNA under 200 mM salinity in both the leaf and root tissues (S3 and S4 Tables).

*CFB3* was up-regulated in all the varieties in the leaf and root tissues under both 100 mM and 200 mM NaCl stress. At 100 mM salinity level, up-regulation of *CBF3* in the leaf tissue was the highest in Q (2.5-fold), whereas under 200 mM NaCl stress condition it was highest in SZ (3.3-fold) in comparison to the control. In BS and KA, *CBF3* mRNA content was slightly lower in 200 mM compared to 100 mM salinity. Similarly, *CBF4* transcript was up-regulated in the leaf and root tissues of all the varieties under both levels of salinity. The mRNA level of all varieties increased with increasing salinity level in both leaf and root tissues except SZ, where its expression was reduced in the leaf at 200 mM NaCl than at 100 mM NaCl. Fold-change expression of the genes under drought and salinity for all genes are provided as S1, S2, S3 and S4 Tables,and heatmaps as S4 and S5 Figs.

## Discussion

ABA accumulates under osmotic stress and other water stresses. It mediates stress responses in vegetative tissues in plant at both genetic and biochemical levels, although not all stress responses are ABA-dependent [40–43]. Expression pattern of ABA-responsive genes is regulated mainly by two different family of bZIP TFs: one that is active in seeds (ABIs) and the other in vegetative tissues (AREB/ABFs) [6][44–47]. ABFs are generally highly conserved although the conserved regions do not have easily recognizable motifs. *AREB/ABF* genes are active in two different ways: they produce functional proteins to stabilize the plant cells and interact with other regulatory proteins under stress conditions; and they play a central role by modulating expression of downstream genes directly or indirectly through ABA [12]. *ABFs* may also be involved in nuclear translocation and transcriptional activation [48]. Each *ABF* gene may function in different ABA-dependent stress signaling pathways. Each AREB/ABF protein may play a specific role in addition to the shared roles with other AREB/ABFs. Although all AREB/ABFs are ABA-inducible and can bind to same ABREs, they are induced by various stress treatments including ABA, and their expression patterns are different from each other in a tissue-dependent manner [48]. The difference in their expression level in response to different stress conditions may be related to the roles of each *AREB/ABF* and to the list of their target genes [15][49].

In the present study *AREB1* expression was up-regulated under both drought and high salt stress, but *AREB2* was more responsive to salt stress than drought stress. Both *ABF1* and *ABF2* were overexpressed under both drought and salinity stresses, but *ABF2* expression was enhanced mostly under drought stress condition. Boneh et al. [11] and Choi et al. [48] showed that the expression of *AREB1/ABF2* and *ABF3* was induced by high salt, and *AREB2/ABF4* was induced by cold, high salt and drought in *Arabidopsis*, which indicated that *AREB1/ABF2* and *ABF3* are involved in high salt signal transduction, whereas *AREB2/ABF4* participates in multiple stress responses [48]. Yoshida et al. [12] also showed that *AREB1*, *AREB2*, and *ABF3* were up-regulated by water stress and ABA. Also, all these three *AREB/ABF* TFs required ABA for full activation in both *Arabidopsis* and rice. These studies and the present study clearly suggested that *AREB/ABF* genes have largely overlapping functions. *Arabidopsis* mutants for each of these genes showed reduced drought tolerance, whereas a triple mutant was more resistant to ABA and sensitive to drought stress. Several other studies reported that *AREB/ABF*-overexpressing plants showed ABA hypersensitivity and enhanced tolerance to abiotic stresses, such as freezing, drought and salt stress [15][49–51]. The present results showed that *AREB/ABFs* had different expression levels in different tissues in grape in response to drought and salinity stress. Boneh et al. [11] reported that the stress and ABA-regulated expression of the *ABF* genes were organ-specific in grape. In grape, two putative ABFs were reported to interact with SnRK2, which is upstream of the phosphorylation cascade of ABA double-negative regulatory mechanism, and thus phosphorylated ABF to its functionally active form [11].

The CBF/DREB proteins play a critical role in abiotic stress-mediated gene expression and represent one of the most attractive regulons for breeding programs. Most *CBF* genes are known to be induced/up-regulated in response to cold but not drought stress [29][31]. However, several studies showed that *DREB/CBF* genes are induced by various stresses, and that ABA is capable of activating some *DREB/CBF* genes [29][31] [52][53]. Thirty eight VvDREB members, organized into A1 through A6 subgroups of *Arabidopsis* DREBs, were identified from the entire grapevine genome and its expression sequence tag assembly [54]. In the present study, *CBF1* and *CBF3* were expressed more under salinity, whereas *CBF2* was responsive to both drought and high salinity stress. Up-regulation of *CBF4* was observed under both drought and salinity stresses in the leaf and root tissues of grape. In *Arabidopsis*, *AtCBF4* was induced by drought but not cold [27][31][32]. *CBF4* is known to be more responsive to osmotic stress in plants and was induced by exogenous ABA application in *Arabidopsis* and grape [27][29]. Expression of *CBF4* in response to cold stress could be because of osmotic stress [29]. In *Medicago*, it was expressed in response to cold, drought and high salt stresses, and ABA treatment [55]. *CBF1-3* transcript level also increased in response to elevated ABA levels, which was partly due to the increased activity of CBF promoters in response to ABA [27][52]. In the present study, *CBF* genes were most responsive to drought stress, although they were expressed at high level under salt stress. Overexpression of *CBF* genes enhanced drought and salt tolerance in *Arabidopsis*, rice and *Medicago* [55][56]. Evaluation of the expression patterns of three nucleus-localized *DREB/CBF* (*CBF1*, *CBF2* and *CBF3*) genes in grape showed that their transcripts were more in young tissues than in old tissues [28]. Varying amounts of *CBF1* transcript observed in grape could be explained by the plant condition that influenced its stress response to stress or could be because of plant cells memory to the previously encountered stress [52][57]. Further, DREB was shown to interact with AREB in ABA-induced slow gene expression in vegetative tissues under dehydration stress conditions [58]. In the present study, varietal difference in the amounts of *ABF* and *CBF* transcripts was observed even under unstressed control conditions, although no significant difference was observed in the expression of the *CBF* genes between *V*. *vinifera* and *V*. *riparia* [28]. High levels of these TFs were observed in stress tolerant varieties in comparison to the sensitive variety. Based on the phenotype data (S1 Fig) supported by the gene expression results, varieties SZ, KA and Q were considered resistant to drought and salinity stresses; KA appeared to be the most tolerant variety to salt stress. In all of these tolerant varieties a high level of expression of *AREB/ABF* and *DREB/CBF* genes was observed. High level of expression of these TFs under the control condition in the resistant varieties is an indicative of their anticipatory preparedness to respond better to a particular stress condition compared to the susceptible variety. DREB proteins can function with AREB in the expression of dehydration responsive *rd29A* gene under high-salinity conditions. Therefore, further detail studies are needed especially in resistant varieties of grape to unravel interaction between the AREB/ABRE and DREB/DRE regulatory systems that modulates expression of downstream stress responsive genes in response to ABA accumulated under drought and high-salinity conditions. Further validation of the expression of these TFs in a diverse collection of grape varieties will facilitate their use as candidate expression markers in breeding programs to screen drought and/or salinity resistant/susceptible genotypes.

## Supporting Information

S1 Fig Drought (a) and salinity (200 mM; b) stress response of grape varieties. Q = Qalati, SA = Sabz Angoor, SZ = Sabz angoor, KA = Kaj angoor, B = Bidaneh sefid; 1 = control, 2 = - 1.5 Mpa drought stress. Picture was taken three weeks and two weeks after drought (a) and salinity (b) stress treatment, respectively.
(PPTX)

S2 Fig Cluster analysis of AREB/ABF candidates from grape with homologs from other plants. (PPTX)

S3 Fig mRNA accumulation of elongation factor *VvElf1A* in the leaf and root tissues under drought and salt stresses of grape varieties. (PPTX)

S4 Fig Heat map showing expression pattern of genes in the leaf (left panel) and root (right panel) tissues under drought stress in grape. T1, -0.3 Mpa; T2, -0.7 Mpa; T3, -1.0 Mpa; T4, -1,5 Mpa.
(PPTX)

S5 Fig Heat map showing expression pattern of genes in the leaf (left panel) and root (right panel) tissues under salinity stress in grape. T1, control; T2, 100 mM NaCl; T3, 200 mM NaCl.
(PPTX)

S1 Table Fold changes in the expression of genes under drought in the leaf tissues of grape varieties. (DOCX)

S2 Table Fold changes in the expression of genes under drought in the root tissues of grape varieties. (DOCX)

S3 Table Fold changes in the expression of genes under salt stress in the leaf tissues of grape varieties. (DOCX)

S4 Table Fold changes in the expression of genes under salt stress in the root tissues of grape varieties. (DOCX)
