# Supplementary material for: Analyzing the Expression Profile of AREB/ABF and DREB/CBF Genes under Drought and Salinity Stresses in Grape (Vitis vinifera L.)
Source: PLoS One. 2015 Jul 31;10(7):e0134288. doi: 10.1371/journal.pone.0134288 (PMC4521911; doi:10.1371/journal.pone.0134288)
Supplement: S1 Fig — Q = Qalati, SA = Sabz Angoor, SZ = Sabz angoor, KA = Kaj angoor, B = Bidaneh sefid; 1 = control, 2 = - 1.5 Mpa drought stress. Picture was taken three weeks and two weeks after drought (a) and salinity (b) stress treatment, respectively. (PPTX) [file pone.0134288.s001.pptx]

## Slide 1
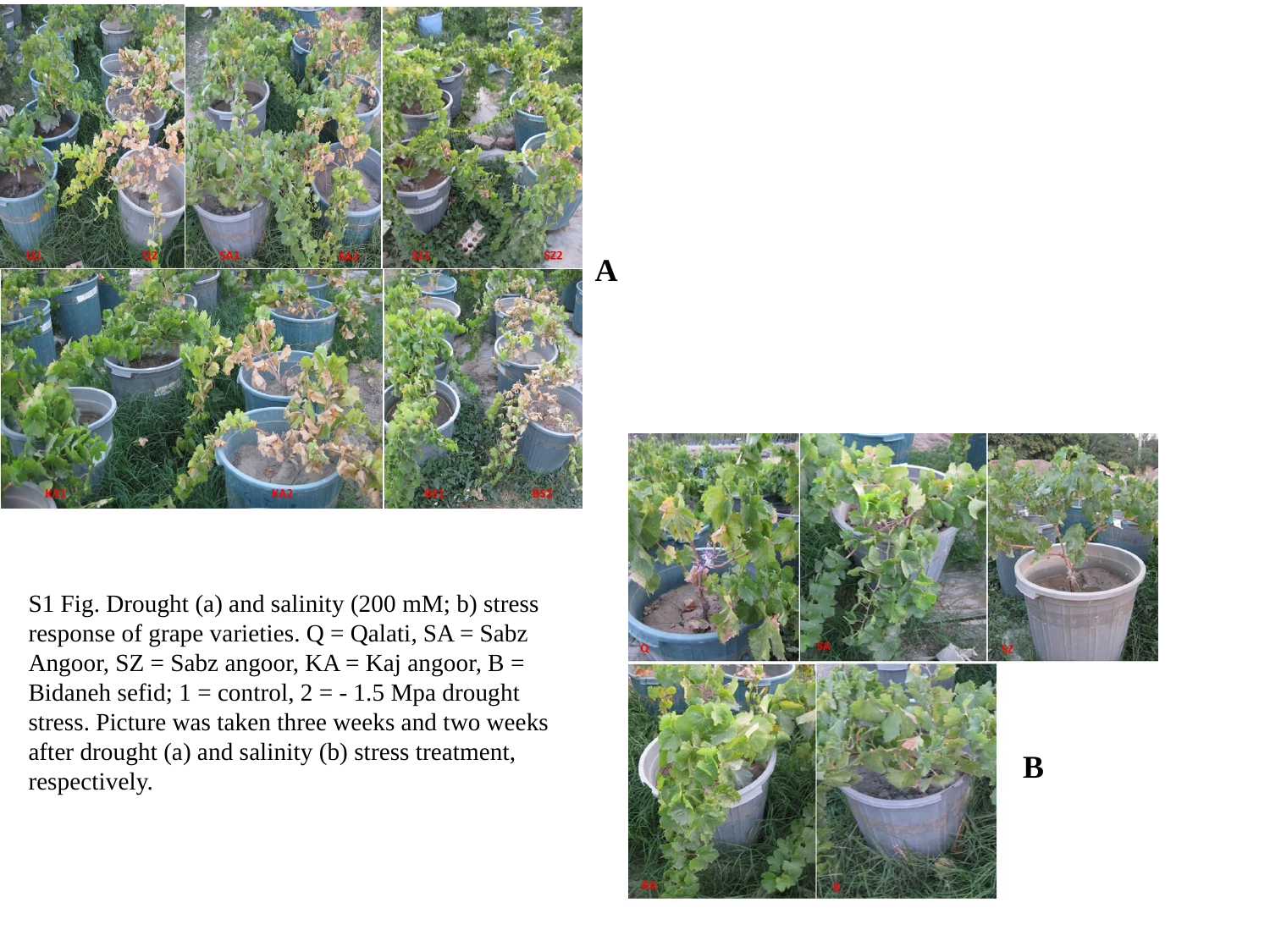

A
S1 Fig. Drought (a) and salinity (200 mM; b) stress response of grape varieties. Q = Qalati, SA = Sabz Angoor, SZ = Sabz angoor, KA = Kaj angoor, B = Bidaneh sefid; 1 = control, 2 = - 1.5 Mpa drought stress. Picture was taken three weeks and two weeks after drought (a) and salinity (b) stress treatment, respectively.
B
