# Supplementary figures and images for: Analyzing the Expression Profile of AREB/ABF and DREB/CBF Genes under Drought and Salinity Stresses in Grape (Vitis vinifera L.)
Source: PLoS One. 2015 Jul 31;10(7):e0134288. doi: 10.1371/journal.pone.0134288 (PMC4521911; doi:10.1371/journal.pone.0134288)

## Slide 1
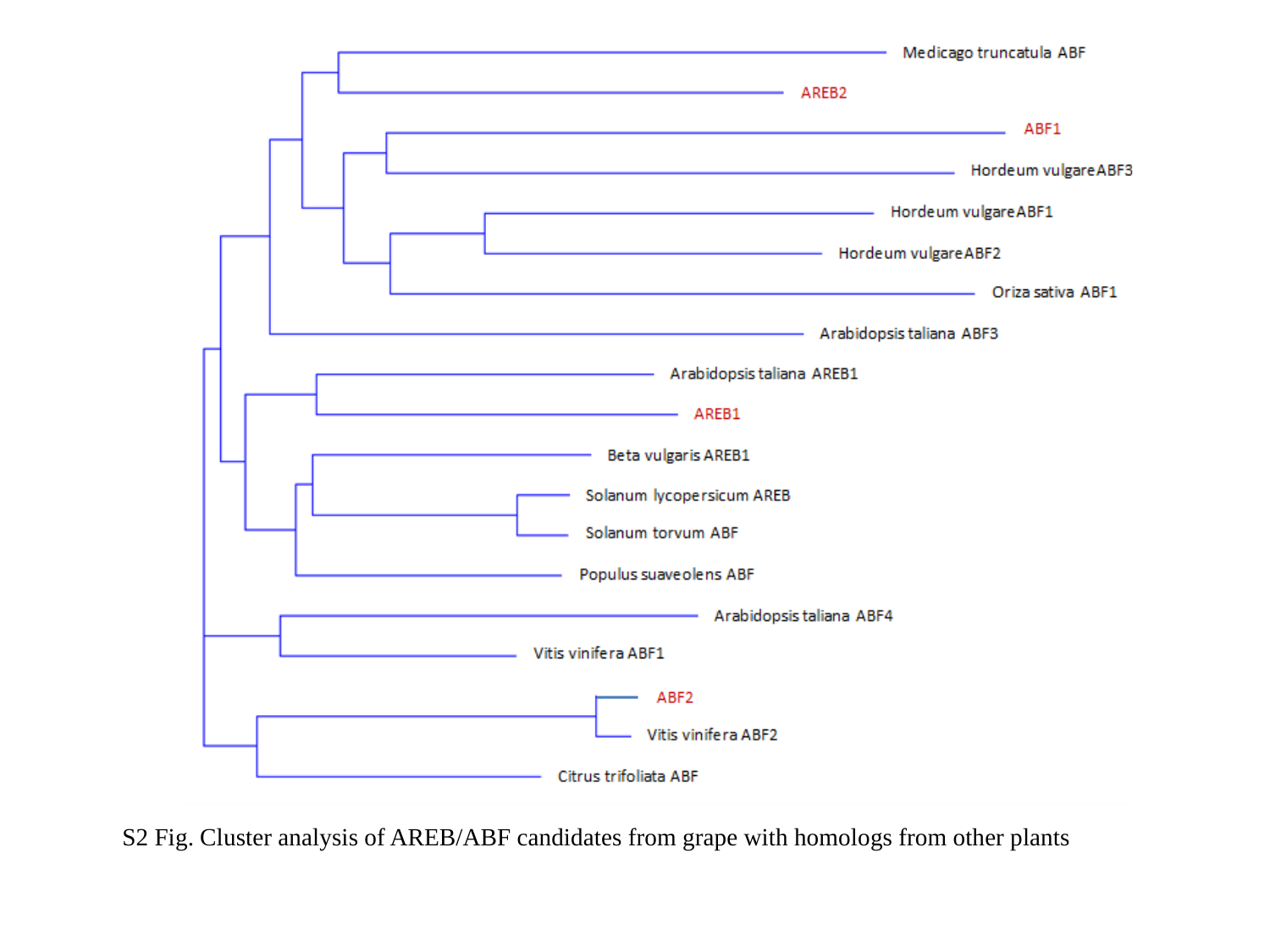

S2 Fig. Cluster analysis of AREB/ABF candidates from grape with homologs from other plants

Supplement: S2 Fig — (PPTX) [file pone.0134288.s002.pptx]
