# Supplementary material for: Analyzing the Expression Profile of AREB/ABF and DREB/CBF Genes under Drought and Salinity Stresses in Grape (Vitis vinifera L.)
Source: PLoS One. 2015 Jul 31;10(7):e0134288. doi: 10.1371/journal.pone.0134288 (PMC4521911; doi:10.1371/journal.pone.0134288)
Supplement: S3 Fig — (PPTX) [file pone.0134288.s003.pptx]

## Slide 1
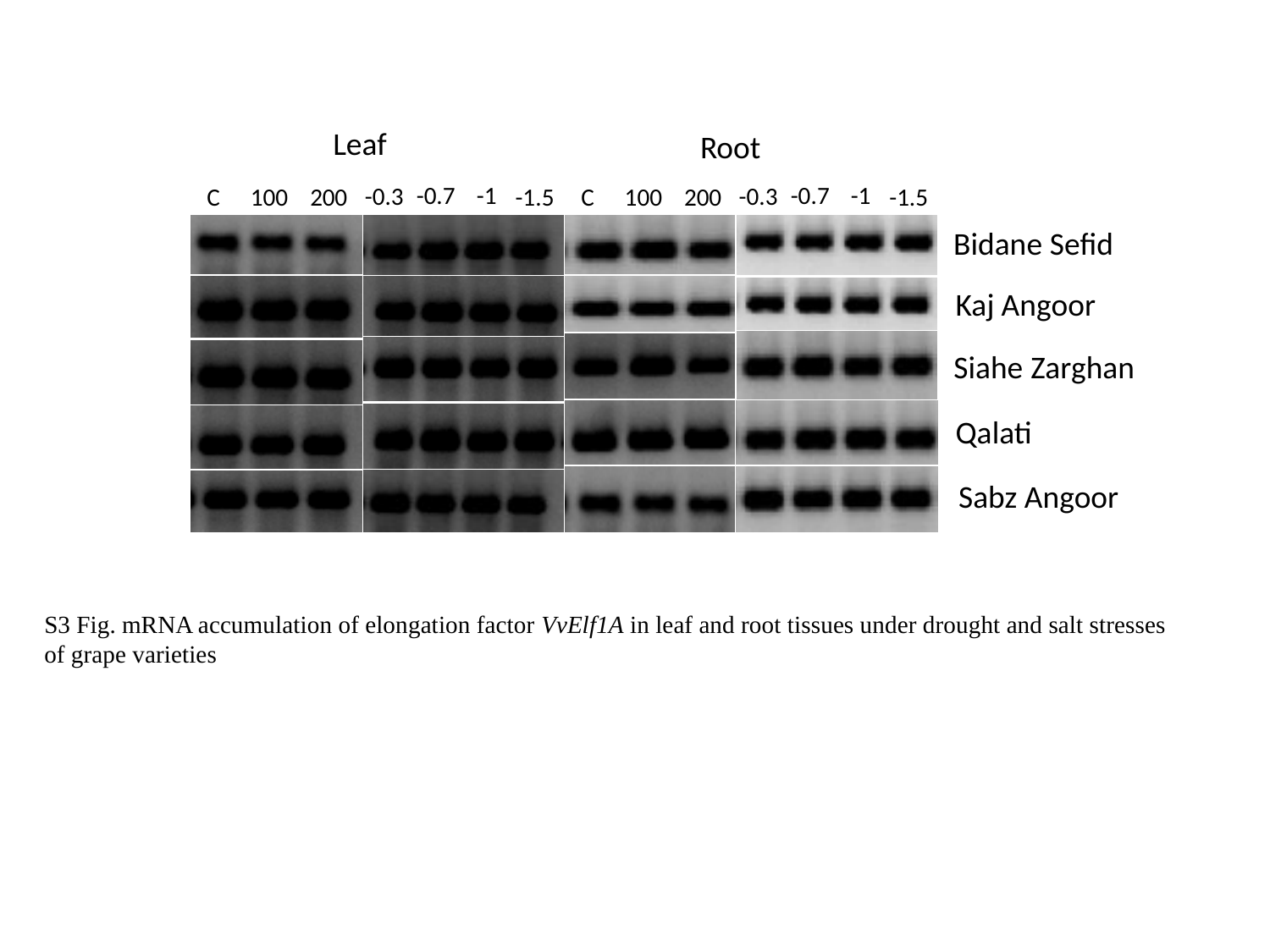

S3 Fig. mRNA accumulation of elongation factor VvElf1A in leaf and root tissues under drought and salt stresses
of grape varieties
