# Supplementary material for: Analyzing the Expression Profile of AREB/ABF and DREB/CBF Genes under Drought and Salinity Stresses in Grape (Vitis vinifera L.)
Source: PLoS One. 2015 Jul 31;10(7):e0134288. doi: 10.1371/journal.pone.0134288 (PMC4521911; doi:10.1371/journal.pone.0134288)
Supplement: S4 Fig — T1, -0.3 Mpa; T2, -0.7 Mpa; T3, -1.0 Mpa; T4, -1,5 Mpa. (PPTX) [file pone.0134288.s004.pptx]

## Slide 1
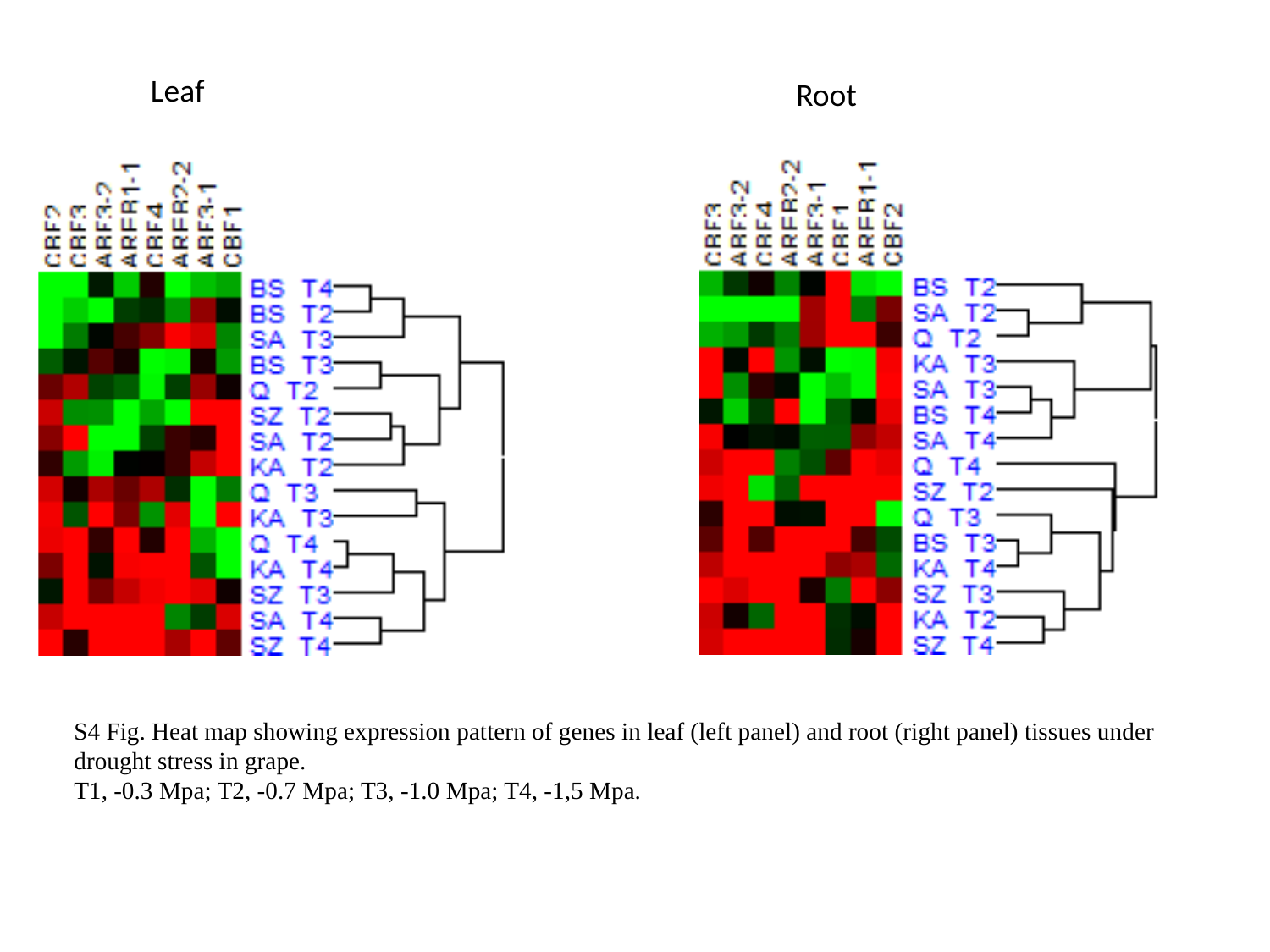

Leaf
Root
S4 Fig. Heat map showing expression pattern of genes in leaf (left panel) and root (right panel) tissues under drought stress in grape.
T1, -0.3 Mpa; T2, -0.7 Mpa; T3, -1.0 Mpa; T4, -1,5 Mpa.
