# Supplementary material for: Analyzing the Expression Profile of AREB/ABF and DREB/CBF Genes under Drought and Salinity Stresses in Grape (Vitis vinifera L.)
Source: PLoS One. 2015 Jul 31;10(7):e0134288. doi: 10.1371/journal.pone.0134288 (PMC4521911; doi:10.1371/journal.pone.0134288)
Supplement: S1 Table — (DOCX) [file pone.0134288.s006.docx]

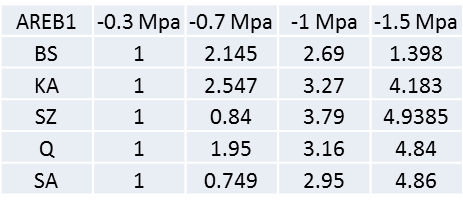

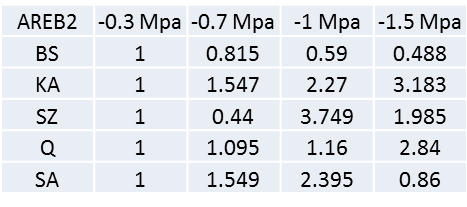

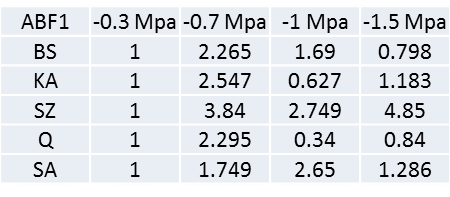

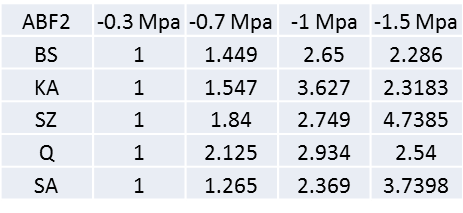

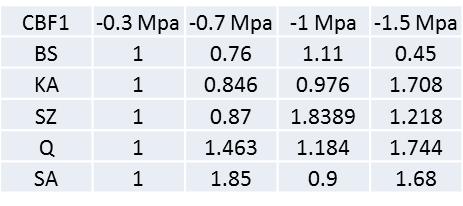

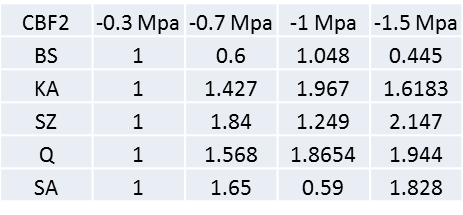

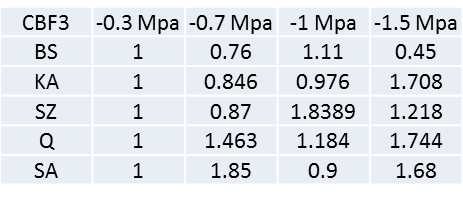

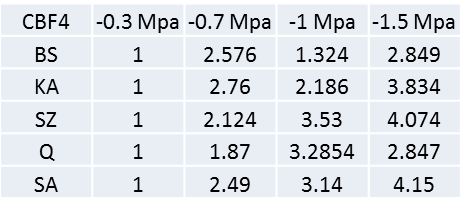


S1 Table. Fold changes in the expression of genes under drought in the leaf tissues of grape varieties
